# Histopathologic Reassessment of Placental Vascular Lesions Based on the Amsterdam Consensus Criteria: A Retrospective Analysis of 571 Placental Cases

**DOI:** 10.3390/medicina62040773

**Published:** 2026-04-16

**Authors:** Büşra Altunay Ünal, Esra Çobankent Aytekin, Havva Serap Toru

**Affiliations:** 1 Department of Pathology, Kütahya City Hospital, 43100 Kütahya, Türkiye; busraaltunay9@gmail.com; 2 Department of Pathology, Konya Numune Hospital, 42060 Konya, Türkiye; esracobankent@hotmail.com; 3 Department of Pathology, Faculty of Medicine, Akdeniz University, 07058 Antalya, Türkiye

**Keywords:** placenta, placental pathology, maternal vascular malperfusion, fetal vascular malperfusion, chorangiosis, placental vascular lesions, Amsterdam consensus

## Abstract

*Background and Objectives*: Placental vascular lesions are significant histopathological findings that indicate disruptions in uteroplacental and fetoplacental circulations and are associated with adverse pregnancy outcomes such as preeclampsia, intrauterine growth restriction (IUGR), and perinatal morbidity. This study aimed to re-examine the frequency and distribution of placental vascular lesions in placentas submitted for histopathological analysis at our center, based on criteria established by the Amsterdam Placental Workshop Group Consensus Statement. *Materials and Methods*: In this retrospective study, placental samples examined in the Department of Pathology at Akdeniz University Faculty of Medicine from 2016 to 2019 were analyzed. A total of 571 cases with at least one placental vascular lesion identified on histopathology were included. Hematoxylin–eosin-stained sections from all cases were re-evaluated, and maternal vascular malperfusion (MVM), fetal vascular malperfusion (FVM), and other placental vascular pathologies were assessed according to the Amsterdam consensus criteria. Statistical analyses were performed using IBM SPSS Statistics for Windows, Version 25 (IBM Corp., Armonk, NY, USA). Categorical variables were compared using the chi-square or Fisher’s exact test, while continuous variables were analyzed with the Mann–Whitney U test. *Results*: MVM and FVM were considered the primary outcomes of the study. MVM was identified in 95.1% of cases, whereas FVM was present in 1.9%. Among individual lesions, chorangiosis (97.2%) and villous/perivillous fibrinoid deposition (88.3%) were the most frequent findings, followed by mucinous cystic degeneration of the umbilical cord (61.5%) and dystrophic calcification (58.1%). Retroplacental hematoma was observed in 38.4% of cases. Although no significant association was found between MVM and placental weight or size, umbilical cord length was significantly shorter in MVM-positive cases (*p* = 0.032). In contrast, FVM showed significant associations with chorangiosis (*p* = 0.035) and placentomegaly (*p* = 0.003). The high frequency of chorangiosis may reflect a compensatory angiogenic response to chronic intrauterine hypoxia, potentially mediated by vascular growth factors, with variable effectiveness depending on the severity of the underlying condition. *Conclusions*: These findings suggest that placental vascular lesions are not only markers of obstetric complications but also serve as morphological indicators of fetoplacental adaptive responses.

## 1. Introduction

The placenta is a multifunctional organ that plays a vital role in supporting pregnancy by enabling the exchange of oxygen and carbon dioxide between maternal and fetal blood, transferring nutrients and electrolytes, and helping to maintain fetomaternal balance through its hormonal and immune functions. As a result, structural or circulatory problems of the placenta can negatively affect not only the progression of pregnancy but also fetal growth and perinatal health outcomes. Placental vascular lesions caused by impaired uteroplacental or fetoplacental blood flow are associated with adverse pregnancy outcomes such as preeclampsia, intrauterine growth restriction (IUGR), preterm birth, and stillbirth [1,2].

For many years, inconsistencies in terminology and sampling methods in placental pathology have hindered the evaluation of clinicopathological correlations and the comparability of studies. To address this issue, the Amsterdam Placental Workshop Group Consensus Statement established standardized criteria for defining and classifying placental lesions, especially MVM and FVM, and has become a key reference in modern placental pathology practice [3]. In this study, placental vascular lesions were classified according to the Amsterdam Placental Workshop Group Consensus Statement (2016), which defines standardized criteria for MVM and FVM, enabling consistent histopathological evaluation and clinicopathological correlation.

MVM indicates a placental injury pattern caused by impaired uteroplacental perfusion and is closely associated with obstetric complications such as preeclampsia and IUGR. The underlying pathophysiological mechanism involves insufficient remodeling of spiral arteries and defective deep placentation, leading to decreased uteroplacental blood flow [1]. According to the Amsterdam criteria, the spectrum of MVM includes histopathological findings such as distal villous hypoplasia, accelerated villous maturation, increased syncytial knotting, and placental infarction [3,4].

FVM signifies inadequate perfusion of the villous parenchyma by fetal blood due to disruptions in the fetoplacental circulation, usually caused by thrombotic or obstructive processes affecting the fetoplacental vascular bed. Histopathologically, it is characterized by avascular villi and villous stromal–vascular karyorrhexis. Severe FVM lesions have been associated with neonatal neurological morbidity [3,5].

In addition to MVM and FVM, placental vascular lesions can also appear as villous capillary abnormalities, including chorangiosis, chorangioma, and chorangiomatosis. These are characterized by capillary proliferation within the chorionic villi.

Chorangiosis is defined by increased capillary density in terminal villi and has been associated with chronic intrauterine hypoxia and adverse perinatal outcomes such as IUGR, preterm birth, and stillbirth [6,7].

Normal histological changes such as intervillous fibrin deposition and dystrophic calcifications may be observed in term placentas; however, their extent and distribution are essential in distinguishing physiological findings from pathological lesions, as emphasized in the Amsterdam consensus criteria.

Chorangioma, the most common benign vascular tumor of the placenta, can lead to pregnancy complications such as polyhydramnios and non-immune fetal hydrops, especially when lesions measure ≥4 cm due to significant arteriovenous shunting [8].

Placental mesenchymal dysplasia (PMD) is a rare vascular anomaly marked by placentomegaly and mesenchymal hyperplasia of stem villi. PMD has been reported to be linked with intrauterine growth restriction in affected pregnancies [9].

Although there is increasing recognition of the clinical significance of placental vascular lesions, large-scale histopathological studies that evaluate their frequency and distribution using standardized criteria remain scarce. In this study, placentas submitted for histopathological examination at our institution were re-evaluated with the Amsterdam Placental Workshop Group criteria, and the prevalence and distribution of placental vascular lesions were analyzed. To the best of our knowledge, this study is the first comprehensive evaluation in the English literature to re-assess histopathologically various placental vascular pathologies—including chorangiosis, PMD, retroplacental hematoma, and chorangioma—within a large cohort using standardized diagnostic criteria. Since most previous studies have examined these lesions either in isolation or within specific clinical contexts, the integrated analysis of multiple vascular pathologies within the same cohort may help fill an important gap in the literature.

## 2. Materials and Methods

### 2.1. Study Design and Case Selection

This retrospective study examined placental specimens histopathologically at the Department of Pathology, Akdeniz University Faculty of Medicine, from 2016 to 2019. A total of 2173 placentas were screened during this period. Cases submitted to the pathology laboratory that showed at least one placental vascular lesion on histopathological examination were included.

The study group consisted of 571 placental cases, all demonstrating at least one placental vascular lesion linked to maternal and/or fetal circulatory disturbances. Placentas without histopathological vascular lesions were excluded from the study.

During macroscopic examination, placental weight and dimensions, the presence of retroplacental hematoma, thick-walled and/or tortuous vascular structures, and grape-like areas were assessed. Additionally, umbilical cord length was recorded. For histopathological evaluation, two peripheral and two central placental samples, three umbilical cord samples, and fetal membrane samples were collected in all cases. Moreover, the pregnancy status (singleton or multiple gestation) was also documented. Complete data for all these parameters were available for every case.

### 2.2. Histopathological Evaluation

All placental specimens were routinely fixed in 10% neutral buffered formalin and embedded in paraffin blocks. Hematoxylin–eosin (H&E)-stained sections were re-evaluated under a light microscope.

During histopathological examination, various abnormalities of the placenta and umbilical cord were evaluated, including MVM, FVM, placental infarction, avascular villi, chorangiosis, dystrophic calcification, retroplacental hematoma, hydropic and sclerotic villous structures, villitis/intervillitis, PMD, fibrinoid deposition, increased syncytial knots, chorangioma, amniotic fluid infection sequences, Hofbauer cell proliferation, umbilical cord hemorrhage, funisitis, bilobed placenta, funicular hematoma, mucinous cystic changes, and cystic villous alterations.

Placental vascular lesions were classified as MVM and FVM according to the standardized diagnostic criteria outlined in the Amsterdam Placental Workshop Group Consensus Statement [3].

MVM was evaluated based on the presence of villous and maternal vascular lesions indicating uteroplacental perfusion insufficiency, including distal villous hypoplasia, accelerated villous maturation, increased syncytial knots, placental infarction, retroplacental hematoma, and fibrinoid necrosis. Cases with at least one of these findings were considered positive for MVM.

Histopathological findings indicating fetoplacental circulatory issues, such as avascular villi, villous stromal–vascular karyorrhexis, thrombosed fetal vessels, stem villous vascular obliteration, and villous stromal fibrosis, were evaluated; the presence of at least one of these lesions was considered evidence of FVM.

**Figure 1:**
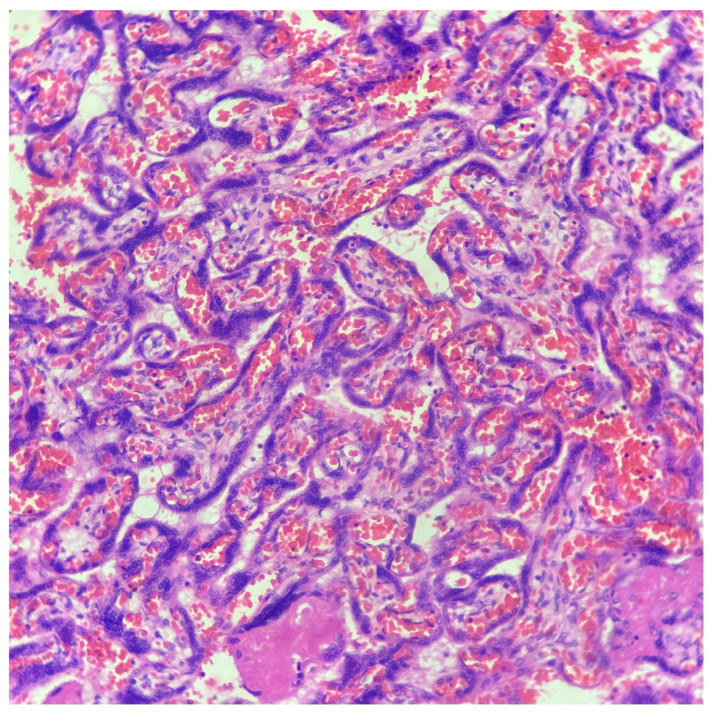
Chorangiosis. Histological appearance of the placenta at 37 weeks of gestation. Terminal villi show increased capillarization with features consistent with chorangiosis. The villous stroma appears mildly edematous, and intervillous spaces contain abundant erythrocytes. Hematoxylin and eosin (H&E) stain.

**Figure 2:**
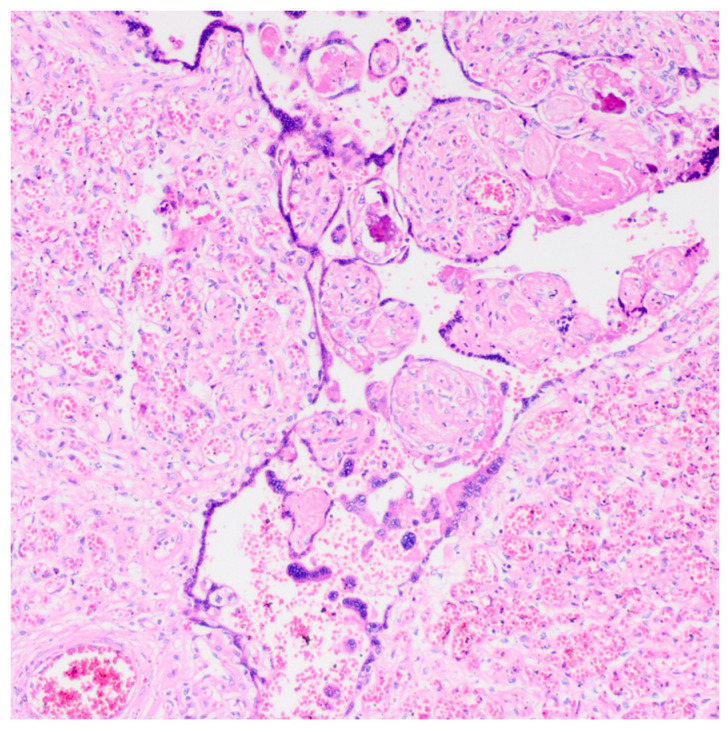
Chorangioma. Histopathological image of the placenta at 38 weeks of gestation showing a well-circumscribed nodular lesion composed of proliferating capillary-sized vascular channels within the chorionic villi, consistent with chorangioma (H&E stain).

The diagnosis of chorangiosis, characterized by villous capillary proliferation, was made based on the Altshuler criteria [7] (Figure 1). The diagnosis of chorangioma was identified by the presence of well-defined vascular lesions showing capillary proliferation within the villous stroma [3] (Figure 2).

**Figure 3:**
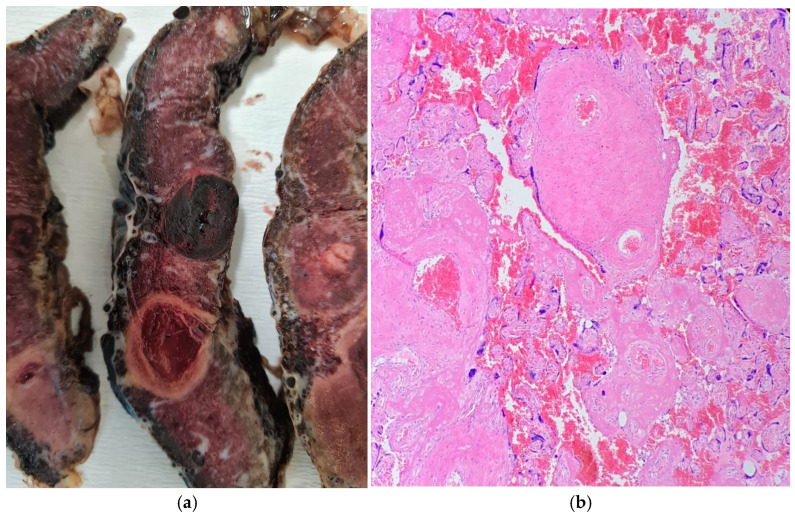
Placental mesenchymal dysplasia (PMD). (**a**) Macroscopic appearance of the placenta at 30 weeks of gestation showing placentomegaly and multiple cystic, grape-like vesicular structures. (**b**) Microscopic features characterized by enlarged stem villi with hydropic stroma, formation of cistern-like spaces, and abnormally thick-walled vessels, without evidence of trophoblastic proliferation (hematoxylin and eosin stain).

The diagnosis of PMD was made based on the macroscopic and microscopic features described in the literature. During macroscopic examination, placentomegaly, subamniotic or intraplacental hematoma, as well as tortuous, dilated, and varicose chorionic vessels and vesicular or cystic areas were observed [10,11]. In microscopic evaluation, supporting findings for PMD included abnormally enlarged stem villi, hypercellular stromal tissue with or without cistern-like spaces, thick-walled villous vessels, and the absence of trophoblastic proliferation [11]. In Mosaic, loss of p57 expression in the dysplastic villous stroma was accepted as an immunohistochemical marker supporting the diagnosis [12]. In cases where pregnancy was terminated before the 20th week of gestation, assessment was conducted solely based on microscopic criteria (Figure 3).

Placentomegaly was defined as a placental weight above the 90th percentile for gestational age, while placental hypoplasia was defined as a placental weight below the 10th percentile [13].

**Figure 4:**
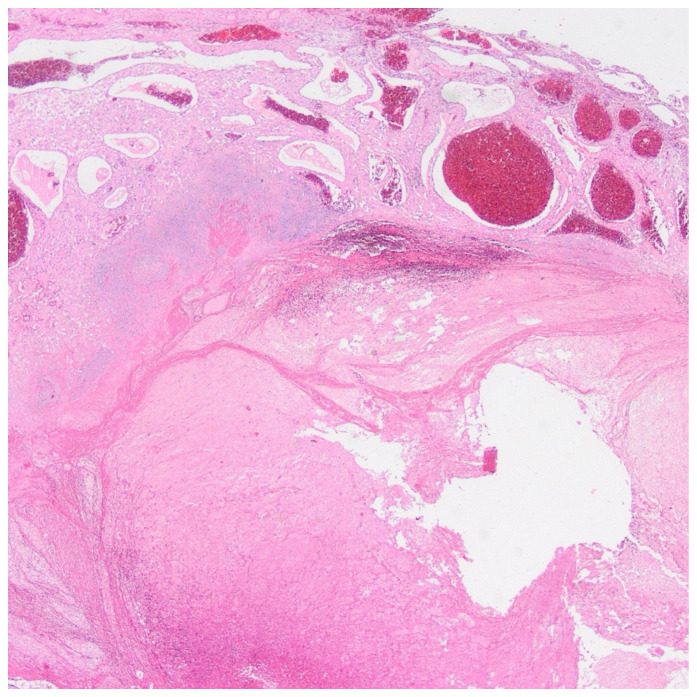
Retroplacental hematoma. Histopathological image of the placenta at 36 weeks of gestation showing a large area of hemorrhage and clot formation located between the placental parenchyma and the maternal surface, consistent with retroplacental hematoma (hematoxylin and eosin stain).

Retroplacental hematoma was identified by visually detecting hemorrhagic areas between the placenta and the uterine wall on the maternal surface [14] (Figure 4).

### 2.3. Statistical Analysis

Statistical analyses were performed using IBM SPSS Statistics for Windows, Version 25.0 (IBM Corp., Armonk, NY, USA).

Continuous variables were presented as mean ± standard deviation and median values, while categorical variables were shown as counts and percentages.

Pearson’s chi-square test was used to compare categorical variables between groups, and Fisher’s exact test was applied when the expected cell count was less than 5.

Since the assumption of a normal distribution was not met, the non-parametric Mann–Whitney U test was used to compare continuous variables based on the presence of MVM and FVM.

A *p*-value less than 0.05 was considered statistically significant for all analyses. Additionally, results with *p* < 0.10 were interpreted as indicating a statistical trend, although they did not reach significance.

### 2.4. Ethics Approval

This study received approval from the Akdeniz University Medical Scientific Research Ethics Committee (Decision No: TBAEK-15, 29 January 2026). All procedures complied with the ethical standards of the institutional research committee and followed the principles of the Declaration of Helsinki.

## 3. Results

A total of 571 placental cases were included in the study, with no missing data for any of the evaluated variables (Table 1). Among these cases, 471 (82.5%) were from the third trimester and 100 (17.5%) from the second trimester. No first-trimester cases were included, as such specimens are typically submitted as curettage material rather than as placental examinations. The distribution of gestational types (singleton and multiple pregnancies) is shown in Table 2. 

The mean placental weight was 594.3 ± 96.5 g, and the median was 590 g. The mean placental diameter was 20.32 ± 3.27 cm, with a median of 18 cm. The mean umbilical cord length was 19.62 ± 8.52 cm, and the median was 19 cm (Table 3).

**Figure 5:**
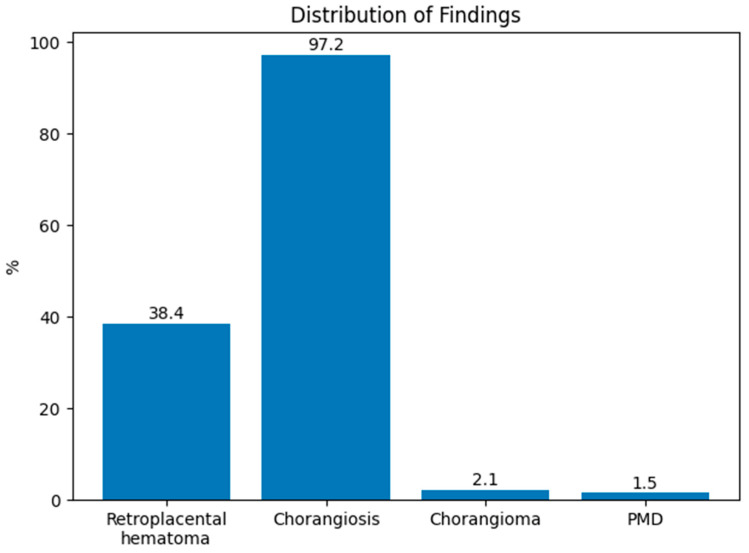
Distribution of placental vascular lesions.

Chorangiosis was observed in 555 cases (97.2%), while retroplacental hematoma was present in 219 cases (38.4%). PMD was detected in 9 cases (1.6%), and chorangioma in 12 cases (2.1%). Placentomegaly occurred in 303 cases (53.1%), and placental hypoplasia was found in 3 cases (0.5%) (Figure 5).

Dystrophic calcification was noted in 332 cases (58.1%), while mucinous cystic degeneration of the umbilical cord was seen in 351 cases (61.5%). An umbilical cord with one artery and one vein was identified in 5 cases (0.9%), and amniotic fluid infection sequence was observed in 33 cases (5.8%).

Avascular and sclerotic villi were found in 2 cases (0.4%), prominent villous and perivillous fibrinoid deposition in 504 cases (88.3%), and bilobed placenta in 1 case (0.2%). Hydropic villi appeared in 35 cases (6.1%), cystic changes in villi in 51 cases (8.9%), and increased Hofbauer cells in 1 case (0.2%). Funicular hematoma was observed in 8 cases (1.4%), and chronic villitis with intervillositis in 2 cases (0.4%). Placental infarction was present in 114 cases (20.0%), while increased syncytial knots were seen in 40 cases (7.0%).

MVM was found in 543 cases (95.1%), while FVM was identified in 11 cases (1.9%).

No statistically significant link was found between the presence of MVM and placental weight or placental diameter (*p* = 0.806 and *p* = 0.213, respectively). However, umbilical cord length was notably shorter in cases with MVM (*p* = 0.032).

A statistically significant link was found between FVM and chorangiosis, with chorangiosis appearing more often in FVM-positive cases (*p* = 0.035). Likewise, a significant connection was seen between FVM and placentomegaly, with placentomegaly being more common in FVM-positive cases (*p* = 0.003).

Although no statistically significant link was found between FVM and hydropic villi (*p* = 0.163), hydropic villous structures appeared more frequently in cases with FVM. Similarly, no significant connection was detected between increased syncytial knots and FVM (*p* = 0.462), although a tendency for increased syncytial knot formation was seen in FVM-positive cases.

Among the cases included in the study, 91.9% (*n* = 525) were singleton pregnancies, while 8.1% (*n* = 46) were multiple pregnancies. Of the multiple pregnancies, 10.8% (*n* = 5) were triplet pregnancies, and 79.2% (*n* = 41) were twin pregnancies.

Among the triplet pregnancies, 60% (*n* = 3) were triamniotic trichorionic, and 40% (*n* = 2) were triamniotic dichorionic. Among the twin pregnancies, 23.9% (*n* = 11) were diamniotic monochorionic, 70.7% (*n* = 29) were diamniotic dichorionic, and 2.1% (*n* = 1) were monoamniotic monochorionic.

When evaluating the relationship between multiple pregnancies and histopathological findings, dystrophic calcification (*p* = 0.024), mucinous cystic changes in the umbilical cord (*p* = 0.015), and fibrinoid deposition (*p* = 0.036) were found to be significantly more common in multiple pregnancies.

However, no statistically significant associations were identified between the presence of retroplacental hematoma and MVM, FVM, placental infarction, chorangiosis, or placentomegaly (all *p* > 0.05).

Similarly, no statistically significant associations were found between multiple pregnancy and placental infarction, maternal vascular malperfusion, chorangiosis, umbilical cord hypo- or hypercoiling, placental hypoplasia, PMD, or chorangioma (all *p* > 0.05).

## 4. Discussion

Placental vascular lesions, especially patterns of MVM and FVM, are recognized as key histopathological markers linked to adverse perinatal outcomes such as preeclampsia, IUGR, preterm birth, and stillbirth [1,15,16]. The main pathophysiological mechanism of maternal vascular malperfusion involves inadequate physiological remodeling of spiral arteries, which normally occurs during pregnancy. This results in decreased uteroplacental circulation and impaired blood flow to the placenta, causing uteroplacental hypoperfusion [1]. Similarly, FVM results from fetoplacental vascular obstruction or thrombosis and has been associated with neonatal neurological morbidity [4,5,17].

Following the consensus statement issued by the Amsterdam Placental Workshop Group in 2016, the evaluation of placental lesions based on standardized criteria has been recommended, establishing consistency in placental pathology terminology. However, the number of large-scale studies adhering to these criteria remains limited [3,18,19]. In this study, re-evaluating 571 cases with placental vascular lesions according to the Amsterdam criteria provides valuable insights into the morphological distribution of these pathologies based on standardized diagnostic standards, adding important data to the existing literature.

The finding that 95.1% of cases showed MVM features is much higher than rates reported in other studies. Ernst reported a prevalence of about 30–40%, which is even lower among high-risk pregnancies [2]. This difference likely occurs because our study included only cases with at least one histopathologically confirmed placental vascular lesion. As a result, the selected study population may explain the higher observed MVM frequency compared to population-based studies [2,15]. Additionally, MVM encompasses a wide range of histopathological features—including distal villous hypoplasia, accelerated villous maturation, and infarction—which could also affect prevalence differences.

Previous research has suggested that maternal vascular malperfusion may be linked to placental growth restriction caused by reduced uteroplacental blood flow, resulting in lower placental weight [14,20,21]. However, our study found no significant connection between MVM presence and placental weight or size. This difference might be due to how the morphological effects of MVM relate to the severity and duration of the clinical condition, as well as other maternal and placental factors that could increase placental weight. Conditions associated with placentomegaly, such as maternal diabetes, the common occurrence of lesions like chorangiosis—which may form as an adaptive response to chronic intrauterine hypoxia—and retroplacental hematomas characterized by blood buildup within the placenta, could obscure potential statistical links between placental weight and MVM.

Our study’s inability to thoroughly analyze obstetric parameters—such as preeclampsia or intrauterine growth restriction, which are established clinical indicators of uteroplacental insufficiency—may have limited the ability to demonstrate links between histopathological findings and macroscopic placental features. Additionally, the varied distribution of lesions within the MVM spectrum, in terms of both chronicity and severity, may have caused different effects on macroscopic parameters across cases.

The discovery that the umbilical cord is notably shorter in cases with MVM aligns with previous research, which supports the link between chronic intrauterine hypoxia and reduced fetal movement. Both experimental and clinical data demonstrate that umbilical cord length correlates with fetal activity, and decreased fetal movement under chronic intrauterine stress may result in a shorter cord [22,23]. In this context, the association between MVM and shorter cord length could serve as an indirect marker of uteroplacental perfusion issues. However, in our study, cord length was only measured on the placental side from the segment submitted with the placenta, leaving the portion remaining with the fetus unmeasured. Consequently, the recorded values might not fully reflect the total cord length, and this limitation should be considered.

The strong link between FVM and chorangiosis (*p* = 0.035) supports earlier research indicating that capillary proliferation within terminal villi develops as an adaptive response to chronic intrauterine hypoxia [6,7,24,25,26]. It can be suggested that, in cases of FVM, the hypoxic environment caused by impaired fetoplacental circulation may promote villous angiogenesis, resulting in chorangiosis.

Similarly, the connection between FVM and placentomegaly (*p* = 0.003) supports earlier findings that increased placental volume can occur in vascular anomalies. PMD has been described as a rare vascular anomaly of the placenta often associated with placentomegaly [9]. described PMD as a rare vascular anomaly of the placenta often found with placentomegaly. Recent studies emphasize that PMD is often linked to larger placental size, with extensive villous and stem villous changes backing this morphological pattern [9,24,27,28]. Although no significant association was found between hydropic villous structures and FVM, the higher occurrence may suggest stromal adaptive responses to hypoxia.

More common findings, such as dystrophic calcification, mucinous cystic changes in the umbilical cord, and fibrinoid deposits in multiple pregnancies, may be related to increased placental hemodynamic load and metabolic demands. Previous studies indicate that, due to higher demands in multiple pregnancies, the placenta undergoes more pronounced adaptive and degenerative structural changes.

## 5. Conclusions

In conclusion, this study offers valuable data on the range of placental pathology by reassessing placental vascular lesions in a large series of cases according to the Amsterdam consensus criteria. While some findings align with the current literature, certain differences might be due to the specific characteristics of the study population and the exclusion of clinical obstetric parameters from the analysis.

These findings indicate that placental vascular pathologies might serve not only as outcomes of obstetric complications but also as morphological indicators of fetoplacental adaptive processes. Future prospective studies that include both clinical and histopathological data could enhance understanding of how placental vascular lesions affect perinatal outcomes.

## Figures and Tables

**Table 1:** Distribution of histopathological findings in placental specimens (*n* = 571).

Histopathological Finding	*n*	%
Retroplacental hematoma	219	38.4
Placentomegaly	303	53.1
Placental hypoplasia	3	0.5
Dystrophic calcification	332	58.1
Chorangiosis	555	97.2
Mucinous cystic degeneration of the umbilical cord	351	61.5
Amniotic fluid infection sequence	33	5.8
Avascular and sclerotic villi	2	0.4
Prominent villous and perivillous fibrinoid deposition	504	88.3
Hydropic villi	35	6.1
Cystic changes in villi	51	8.9
Increased Hofbauer cells	1	0.2
Funicular hematoma	8	1.4
Chorangioma	12	2.1
Chronic villitis and intervillositis	2	0.4
Placental infarction	114	20.0
Placental mesenchymal dysplasia	9	1.6
Increased syncytial knots	40	7.0
MVM	543	95.1
FVM	11	1.9

**Table 2:** Distribution of gestational types.

Gestational Type	*n*	%
Singleton pregnancy	525	91.9
Multiple pregnancy	46	8.1
Diamniotic monochorionic twin placenta	11	1.9
Diamniotic dichorionic twin placenta	28	4.9
Monoamniotic monochorionic twin placenta	1	0.2
Triamniotic trichorionic triplet placenta	3	0.5
Triamniotic dichorionic triplet placenta	2	0.4

**Table 3:** Macroscopic placental parameters.

Parameter	Mean ± SD
Placental weight	594.3 ± 96.5 g
Maximum placental diameter	20.32 ± 3.27 cm
Umbilical cord length	19.62 ± 8.52 cm

## Data Availability

The data presented in this study are available on reasonable request from the corresponding author.

## Abbreviations

MVM	Maternal vascular malperfusion
FVM	Fetal vascular malperfusion
IUGR	Intrauterine growth restriction
PMD	Placental mesenchymal dysplasia
H&E	Hematoxylin–eosin
